# How to improve patient retention in an antiretroviral treatment program in Ethiopia: a mixed-methods study

**DOI:** 10.1186/1472-6963-14-45

**Published:** 2014-01-29

**Authors:** Yibeltal Assefa, Lut Lynen, Edwin Wouters, Freya Rasschaert, Koen Peeters, Wim Van Damme

**Affiliations:** 1 Ethiopian Public Health Institute, Addis Ababa, Ethiopia; 2 Department of Clinical Sciences, Institute of Tropical Medicine, Antwerp, Belgium; 3 Department of Social Sciences, Antwerp University, Antwerp, Belgium; 4 Department of Public Health, Institute of Tropical Medicine, Antwerp, Belgium; 5 School of Public Health, University of Western Cape, Cape Town, South Africa

**Keywords:** Antiretroviral treatment, Retention, Attrition, Positive deviance, Framework

## Abstract

**Background:**

Patient retention, defined as continuous engagement of patients in care, is one of the crucial indicators for monitoring and evaluating the performance of antiretroviral treatment (ART) programs. It has been identified that suboptimal patient retention in care is one of the challenges of ART programs in many settings. ART programs have, therefore, been striving hard to identify and implement interventions that improve their suboptimal levels of retention. The objective of this study was to develop a framework for improving patient retention in care based on interventions implemented in health facilities that have achieved higher levels of retention in care.

**Methods:**

A mixed-methods study, based on the *positive deviance approach*, was conducted in Ethiopia in 2011/12. Quantitative data were collected to estimate and compare the levels of retention in care in nine health facilities. Key informant interviews and focus group discussions were conducted to identify a package of interventions implemented in the health facilities with relatively higher or improving levels of retention.

**Results:**

Retention in care in the Ethiopian ART program was found to be variable across health facilities. Among hospitals, the poorest performer had 0.46 (0.35, 0.60) times less retention than the reference; among health centers, the poorest performers had 0.44 (0.28, 0.70) times less retention than the reference. Health facilities with higher and improving patient retention were found to implement a comprehensive package of interventions: (1) retention promoting activities by health facilities, (2) retention promoting activities by community-based organizations, (3) coordination of these activities by case manager(s), and (4) patient information systems by data clerk(s). On the contrary, such interventions were either poorly implemented or did not exist in health facilities with lower retention in care. A framework to improve retention in care was developed based on the evidence found by applying the *positive deviance approach.*

**Conclusion:**

A framework for improving retention in care of patients on ART was developed. We recommend that health facilities implement the framework, monitor and evaluate their levels of retention in care, and, if necessary, adapt the framework to their own contexts.

## Background

The rapid expansion of antiretroviral treatment (ART) is one of the most remarkable achievements in public health history. ART was provided to eight million people by the end of 2011, which is a 20-fold increase since 2003 [1]. In 2011, for the first time, a majority (54%) of people eligible for ART in low- and middle-income countries were receiving the treatment [1].

ART is a life-long intervention that requires a robust framework to adequately monitor and evaluate processes, outcomes and long-term impact, not only at individual patient level but also at health facility and program levels. Patient retention in care is one of the crucial indicators of the success of ART programs [2-7], mainly because high levels of patient retention in care are related to improved adherence to ART, slow progression to AIDS, and increased survival. Moreover, patients who are not retained due to loss to follow-up are likely to develop a high viral load which is associated with an increased risk of infecting other people [8-10]. Hence, countries face the dual challenge of managing and sustaining growing cohorts of patients on ART, in addition to the need for increasing access to ART for the patients who still do not have access to it.

Since the inception of large-scale expansion of ART, ART programs in Africa had retained about 60% of their patients at the end of two years on ART by 2007 [2]. Loss to follow-up was the major cause of attrition, followed by death. Data on the proportion of patients retained on ART over time continue to show that most patient attrition occurs within the first year and that retention rates tend to stabilize thereafter [11]. In 2009, the average global retention rate at 12 months was 82%. It dropped to 77% at 24 months and remained stable at 75% and 74.5% at 36 and 48 months, respectively [3]. These figures are consistent with those from an updated meta-analysis of 39 cohorts from sub-Saharan Africa in 2011 [4]. These findings indicate that retention in care remains to be a challenge for ART programs though it is improving over time [3,4].

Many ART programs have therefore been striving hard to identify and implement appropriate strategies to optimize their retention levels [1]. In addition, it has been identified that levels of retention vary widely across health facilities and programs [3,5]. Hence, health facilities and programs that have achieved higher levels of retention can serve as models for future improvements.

The objectives of this study were to estimate levels of retention in care in nine health facilities in Ethiopia, explain the variability in levels of retention in care across these health facilities, and develop a framework, which will potentially serve as a model, for improving retention. We hypothesized that health facilities with higher and improving retention in care were implementing a number of interventions that positively impact retention.

## Methods

### The antiretroviral treatment program in Ethiopia

A number of initiatives, including resource mobilization, cost reduction, public-private partnerships, and the public health approach, have been undertaken to expand access to ART in Ethiopia [6,10,12]. As a result, ART services have been decentralized and are available in both health centres and hospitals [62]. By mid-2011, more than 333,400 patients were ever started on ART, and 247,800 patients were alive and taking ART. Retention in care has been identified as a real challenge for the ART program in the country; and, hence, a lot of initiatives, including the case management, the peer education and the expert patient programs, have been implemented to improve it [5,13]. These different initiatives have been implemented first as pilot projects since 2007/8, and scaled up in 2009/10. Currently, the case management program is a national initiative launched to be implemented in all health facilities, though its implementation might vary across health facilities.

#### Study design, data collection and analysis

A retrospective cohort study was conducted in 2009 to determine the outcomes of the ART services in 55 health facilities which were selected using a multi-stage random sampling from all regions in the country [12]. Among these 55 health facilities, nine health facilities (one tertiary hospital (HP), two general HPs, two urban health centers (HCs), and four rural HCs), with quite variable levels of cumulative retention in care in 2007/8, were selected purposively and conveniently based on their extreme levels of retention and logistics feasibility, respectively, for an in-depth analysis to identify the reasons for the variability in retention in care across these health facilities.

A mixed-methods study, based on the *positive deviance approach*[14], was conducted in 2011/2012. The *positive deviance approach* is based on the assumption that the solution to a problem can be found within by identifying and learning from organizations and individuals who do their job better than others. The study was conducted in such a way that the rates of retention in care in the nine health facilities were compared to identify health facilities with higher levels of retention in care. Then the package of interventions implemented in the health facilities with higher or improving levels of retention in care, in comparison with interventions in the health facilities with relatively lower levels of retention in care, were explored and compared.

### Quantitative data

*‘Current retention’* in care was the primary outcome we used to compare health facilities for their levels of performance. *‘Current retention’* in care is defined as the retention rate in a specific ‘*calendar’* year among patients who were on ART sometime during the *“calendar”*. The rates of the ‘*current retention’* in care in the nine health facilities were estimated using the tools developed recently to measure retention in care in ART programs [15]. In estimating the *‘current retention’* in care, patients who were lost to follow-up sometime before the ‘*calendar’* year, but traced back and restarted on ART during the ‘*calendar’* year, were included in both the denominator and the numerator.

The rates of retention in care in these health facilities were then compared against a reference to identify health facilities with relatively higher and lower levels of retention in care. FH hospital (HP) and WT health center (HC) were used as references for HPs and HCs, respectively, because of their relatively higher levels of retention in care in 2007/8. The odds of retention in care were then calculated using Epi Info-3.5.1 to check for the significance of the difference in the rates of retention in care in health facilities against the reference health facility. Trends in retention in care were also developed to check if health facilities were improving their levels of retention in care over time. Data were collected from patient registers and individual patient files.

### Qualitative data

Data collection and sampling: Key informant interviews were conducted with service providers to understand the different interventions implemented by the health facilities with better or improving retention in care or by the community-based organizations linked to them. A total of 72 key informants were included in the study until we reached information saturation. The interviewees were clinicians (one to two from each health facility), adherence counselors (one to two from each health facility), case managers (one to two from each health facility), adherence supporters (one to two from each health facility) and community-based service providers (one to two from each community-based organization providing care and support services). The interviewees were purposively selected as key informants since they were thought to have the potential to provide rich, relevant and diverse information pertinent to retention in care and treatment. The interviews were conducted in local language and tape recorded after consent was received. A question guide, focusing on retention in care, was developed and used to facilitate the interview. The guide includes questions related to challenges for and benefits of retention in care, approaches for improving retention in care and interventions implemented to improve retention in care. The guide also asks for the date when these interventions were started to be implemented in the health facility. The key informant interviews were conducted concurrently with the quantitative data collection in such a way that the interviewers and interviewees were blinded to the *‘current retention’* levels of the health facilities under investigation.

A focus group discussion (FGD), with 12 ART mentors to the nine health facilities with better or improving retention in care, was conducted to identify the different interventions implemented by these health facilities in order to improve retention in care. The FGD participants were purposively selected on the basis of their experience in the field and thought to provide rich, relevant and diverse information. A question guide was used to facilitate the discussion. The guide includes questions related to challenges for and benefits of retention in care, theoretical approaches and practical interventions for improving retention in care. The discussion was conducted in local language. It was conducted for one hour and 45 minutes in one of the health facilities with better retention in care in Addis Ababa. It was tape recorded after consent was received from the participants.

The operational definitions of the different variables used for the study are presented in Table 1.

**Table 1:** Operational definitions of the variables related to retention in care

**Variables**	**Definition**	**Numerator**	**Denominator**
**Retention**	All patients who are not registered as deceased or LTFU for any reason	Number of patients alive and on ART	Number of patients alive and on ART plus death plus LTFU
**Loss to follow-up**	Patients who miss scheduled visits to the clinic for more than three months after the last visit	Not applicable (NA)	NA
**Transfer out**	It refers to the official transfer of the patient to another clinic	NA	NA
**Transfer in**	It refers to the official transfer of the patient from another clinic	NA	NA
**Cumulative retention**	The total retention by the end of the calendar among patients ever started on ART	Number of patients alive and on ART by the end of the calendar	The total number of patients ever started on ART
**Current retention**	The retention rate during a specific *“calendar”* among patients who were on ART sometime during the *“calendar”*	Number of patients alive and on ART by the end of the *“calendar”*	Number of patients alive and on ART by the end of the calendar plus number of patients who died plus LTFU during the *“calendar”*
**Calendar**	The time during which the level of the *“current retention”* is estimated	NA	NA

The researchers were all health professionals with experience in HIV/AIDS program management and qualitative and mixed-methods research. The FGD was facilitated by an experienced moderator, the first author, and attended by an observer who took notes.

Data analysis: A concurrent constant comparison was conducted on the field notes and transcripts of the records in line with the question guides used during the interviews and FGD. NVivo version 9 was used to support the qualitative data analysis.

### Ethics statement

This study was approved by the ethical clearance committee of the Ethiopian Health and Nutrition Research Institute. We obtained informed verbal consent from study participants for both conducting and recording the interview. The verbal consent was tape recorded in local languages. We have also got a letter of support from the Federal authorities to collect patient data from the health facilities.

## Results

### Quantitative findings

**Figure 1:**
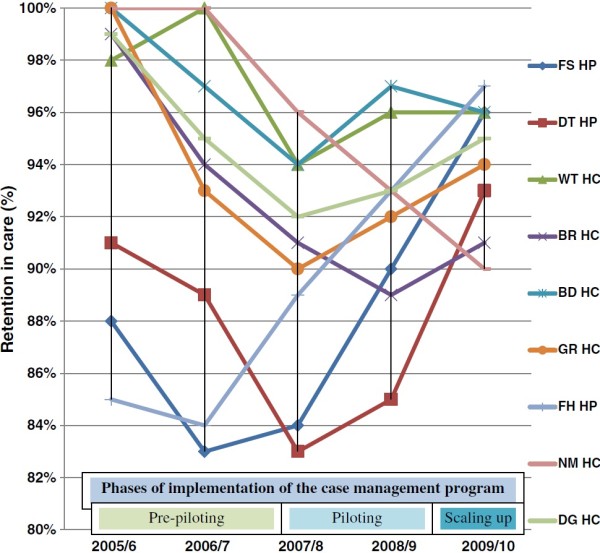
Retention in care in nine health facilities and phases of implementation of the case management program in Ethiopia, 2005/6-2009/10.

The study included health facilities with different levels of care, ranging from tertiary hospital (providing ART to 5629 patients) to rural health centers (providing ART to 577 patients). The median age of patients in the health facilities ranged from 30 to 33 years. The majority of patients were females (ranging from 54% in FH HP to 65% in GR HC) in all health facilities. The median duration that patients were on ART ranged from 14 months in FS HP to 27 months in FH HP. The baseline (at ART initiation) median CD4-cells count ranged from 98 in DT HP to 145 in BR HC. Table 2 and Figure 1 show that all the HCs were receiving patients who were initiated on ART in other health facilities, mainly HPs, and maintaining their care. This was true especially before 2006/7. After 2006/7, these HCs were able to initiate patients on ART in addition to maintaining the care of the patients transferred in. ART delivery was led by physicians in all hospitals and by nurses or health officers in all health centers (Table 2).

**Table 2:** Characteristics of health facilities included in the study, 2009/10

**Characteristics**	**FH HP**	**FS HP**	**DT HP**	**WR HC**	**BR HC**	**BD HC**	**GR HC**	**NM HC**	**DG HC**
**Number of patients on ART**	5629	1062	1159	700	763	859	1299	577	621
**Median age (in years) for patients on ART**	32[27,38]	31[26,39]	32[27,40]	30[26,40]	30[26,39]	31[27,35]	31[27,38]	33[27,40]	31[25,39]
**Female sex for patients on ART**	54%[52,55]	55% [51,59]	56%[53,59]	57%[52,62]	62%[58,67]	55%[51,59]	65%[62,67]	61[55,66]	58%[52,65]
**Median duration on ART (in months)**	27[6,41]	14[6, 26]	16[6,33]	18[8,33]	20[7,33]	17[8,30]	17[6,29]	18[7,30]	18[7,33]
**Median CD4-cells at baseline**	141[71,275]	127[66,190]	98[49, 164]	142[78,206]	157[84,219]	145[85,205]	132[69,194]	106[65,160]	144[84,257]
**Type of health facility**	Tertiary HP	Secondary HP	Secondary HP	Rural HC	Rural HC	Urban HC	Urban HC	Rural HC	Rural HC
Year the health facility started initiating ART*	2003	2005	2005	2006	2006	2006	2006	2007	2007
**ART delivery led by**	Physician	Physician	Physician	Health officer (HO)/Nurse	HO/Nurse	HO/Nurse	HO/Nurse	HO/Nurse	HO/Nurse

*HCs were maintaining the care of patients (before they started initiation) when HPs were transferring out and HCs were transferring in patients.

Table 3 shows that the level of total current retention is variable across hospitals and health centers. FH HP and WT HC had better retention rates compared to other HPs and HCs, respectively. Among HPs, DT HP had the least retention in care (OR = 0.46 (0.35, 0.60), P-value = 0.000); among HCs, BR HC and NM HC had the least retention in care (OR = 0.44 (0.28, 0.70), p-value = 0.000) in 2009/2010. Table 3 also shows that health facilities such as FS HP and DG HC had improved their retention rates over the years. On the other hand, health facilities such as DT HP, BR HC and NM HC had poor retention rates throughout the study period (Table 3).

**Table 3:** **Comparison of ‘*current retention’ *** **in care nine health facilities in Ethiopia, 2007/8-2009/2010**

**Health facility**	**2007/8**			**2008/9**			**2009/10**		
	**Patients retained**	**Patients not retained**	**OR and P-value**	**Patients retained**	**Patients not retained**	**OR and P-value**	**Patients retained**	**Patients not retained**	**OR and P-value**
**FH HP**	4140	490	Reference	4727	368	Reference	5439	190	Reference
**FS HP**	625	116	0.64(0.51,0.80) P-value = 0.000	812	86	0.74(0.57,0.95) P-value = 0.014	1020	42	0.85(0.60,1.21) P-value = 0.343
**DT HP**	863	183	0.56(0.46,0.67) p-value = 0.000	922	168	0.43(0.35,0.52) P-value = 0.000	1077	82	0.46(0.35,0.60) p-value = 0.000
**WT HC**	358	22	Reference	535	20	Reference	669	31	Reference
**BR HC**	474	47	0.62(0.35,1.08) P-value = 0.072	625	78	0.30(0.17,0.51) P-value = 0.000	691	72	0.44(0.28,0.70) p-value = 0.000
**BD HC**	360	24	0.92(0.49,1.74) p-value = 0.79	635	23	1.03(0.54,1.98) p-value = 0.92	821	38	1.00(0.60,1.67) P-value = 0.996
**GR HC**	669	75	0.55(0.32,0.92) p-value = 0.015	964	83	0.43(0.26,0.73) P-value = 0.000	1222	77	0.74(0.47,1.15) p-value = 0.157
**NM HC**	301	13	1.42(0.67,3.04) p-value = 0.32	427	33	0.48(0.26,0.88) p-value = 0.011	522	55	0.44(0.27,0.71) p-value = 0.000
**DG HC**	336	29	0.71(0.39,1.31) p-value = 0.244	463	37	0.47(0.26,0.84) p-value = 0.006	592	29	0.95(0.55,1.64) p-value = 0.833

Figure 1 shows that current retention levels were variable across health facilities, some with relatively high current retention levels and others with relatively low current retention levels. The figure demonstrates that all health facilities except FH HP had a drop in retention in care between 2005/6 and 2007/8. On the contrary, all health facilities except NM HC had been improving their retention between 2007/8 and 2009/10. The performance of NM HC deteriorates quite fast, from the best performer to the worst performer. The variability of retention in care was smaller in 2009/10 (90%-97%) than in 2006/7 (83%-100%). Health facilities such as FS HP and FH HP improved their retention levels remarkably. Figure 1 also depicts the phases of the implementation of the “case management program” in the country. The pre-pilot phase is the phase when there were a number of initiatives implemented in health facilities, such as FH HP and FS HP, to improve retention in care. However, these initiatives were not organized systematically. The initiatives in these health facilities were later structured and organized systematically, and were named “case management program”. This program was piloted in 2007/8. Later, the “case management program” was scaled up and became the national program to improve retention in care.

### Qualitative findings and themes

The qualitative study identified factors that either had contributed to high performance or, when lacking, had created barriers to improvement. Several themes emerged from the interviews and FGDs with service providers in high-and low-performing health facilities.

### Common themes: what relatively high performers have that low performers lack

There is no one factor that easily explains overall performance in any of the health facilities examined. Rather, there are numerous factors that, in combination, appear to contribute to better performance; many are related to or build on each other. Often, the factors that high performers view as essential to improve retention are simply not in place or not considered a priority among the low performers. We identified four themes that contributed to better and improved retention in care in high performers: (1) retention in care promoting activities by health facilities, (2) retention in care promoting activities by community-based organizations, (3) coordination of these activities by case manager(s), and (4) patient information systems by data clerk(s).

#### Retention in care promoting activities by health facilities

Adherence and/or retention-related counseling services were the tasks of all health care providers involved in the delivery of ART in health facilities with better retention in care. Clinicians, adherence counselors (facility-based counselors), pharmacists, case managers (whose task is to coordinate the care of patients), adherence supporters (both facility-based and out reach counselors) and others were all providing counseling services for adherence and retention. All the interviewed providers in health facilities with better retention in care said that counseling was part of their routine task. They added that adherence counseling was very crucial for improving the outcome of patients, and as a result, they had given due emphasis to adherence counseling before and after patients were initiated on ART.

All the interviewed clinicians in health facilities with better retention in care highlighted that poor adherence and retention would have a huge negative effect and thus needed due attention. One of the clinicians said:

“Poor adherence and retention is just like a time-bomb which suddenly blasts and causes a lot of damage to both the patient and the program. This is the main reason that we are working on exploring possible interventions and implementing them to ensure that patients are adherent and retained in care. Hence, adherence and retention is everybody’s work: it is my duty; it is the duty of the data clerk, adherence counselor and the pharmacist; it is, in general, the duty of the multi-disciplinary team. We don’t give it exclusively to one specific cadre. We all work on it, and use every opportunity to make sure that the patient has a positive behavior for adherence and retention.”

We identified, in addition to counseling services, that health facilities with high levels of retention were providing other services such as defaulter tracing and outreach services. Adherence supporters, also called outreach workers, went out from the health facility to the community and trace patients who didn’t show up for their appointment. We found that the cadre called “case manager” was at the center of adherence and/or retention related services. The “case manager” is a lay person who completed high school (twelfth grade), recruited from the community, and trained on adherence and retention for six weeks.

The “case managers” described their role as follows:

“We are responsible to ensure that the patient is getting adequate and holistic care. Clinicians identify patients at risk of poor adherence and/or retention, and send those patients to us. We then assess the level of risk for poor adherence and/or retention of the patient and develop a plan to improve it. Potential reasons (causes) for poor adherence and/or retention are identified. We will accordingly devise appropriate solutions and develop a targeted action plan to reduce the risk of the patient for poor adherence and/or retention. The patient will then be attached with adherence supporters who support him/her towards an improved level of adherence and/or retention. We, the case manager and the adherence supporters, will closely follow and regularly assess the patient for his/her level of adherence and/or retention.”

The “case managers” added: *“if the patient is lost or lost to follow-up in spite of all these efforts, the adherence supporters will either phone or conduct home visits. The adherence supporters will try to bring the patient back to the health facility and restart him/her on treatment. This approach is called “case management” in which the service providers manage cases (beyond diseases) which have both clinical and non-clinical needs.”*

These health facilities also provide care and support services such as home-based care, nutrition and financial support for patients who are bed-ridden and destitute.

#### Retention in care promoting activities by community-based organizations

In several of good performers, community-based organizations including associations of people living with HIV/AIDS played an important role in organizing care and support services for patients taking ART. This concerned mostly adherence counseling, nutrition support, and transportation to and from health facilities, income generating activities, and linkage to other services. Some organizations also provided home-based care services to patients who are bed-ridden and destitute.

A representative of one of the community-based organizations said:

“We understand that patients on ART have a lot of needs. They should have these needs fulfilled directly or indirectly if they have to be adherent to treatment and retained in care. We should thus identify those patients who need support and provide them the necessary support. The mission of our organization is to make sure that patients who are linked to us have got adequate care and support services; we are also doing that”.

Another representative of a community-based organization said:

“Patients have improved their levels of adherence and retention because of the different packages of care and support services we are providing. Services related to adherence and retention are core activities of our organization. We believe these services are working, and we will continue doing them.”

#### Coordination of retention in care promoting activities

This theme summarizes the coordination of activities, by different actors in the continuum of care, intended to improve adherence and/or retention in care. The “case manager” is at the center of the coordination of care provided at the health facility and community levels. The “case manager” with his/her subordinates, expert patients and outreach workers, coordinates the services provided to the patients by the health facilities and community-based organizations.

There are also other coordination mechanisms that have been in place for the care of patients on ART in health facilities with better and improving retention in care: “multi-disciplinary team meetings” and “catchment-area meetings”. “Multi-disciplinary team meetings” involve the different service providers, including clinicians, adherence counselors, pharmacists, case managers, adherence supporters and lab technologists, at health facility level. They discuss about patients who are either at risk of adherence and/or retention, or lost to follow-up or dead. These meetings are conducted regularly and attended by all the different providers in the health facility. “Catchment area meetings” are conducted among health facilities, located in one catchment area as defined by the administration, and community-based organizations. The participants discuss about patients who are lost to follow-up, dead, transfer in or transfer out within the catchment area. These meetings are conducted regularly and attended by all the health facilities and community-based organizations in the catchment area.

One of the participants of the FGD said:

“‘Multi-disciplinary team meetings’ are very crucial in identifying and monitoring the care of patients who are at risk of poor adherence and/or retention. We regularly meet and discuss on patient outcomes. It is a result-oriented meeting focusing on outcomes such as lost to follow-up and death. We also discuss about patients who are potentially at risk of poor adherence and/or retention.”

Another participant of the FGD said:

“Health facilities conducting ‘multi-disciplinary team meetings’ regularly have better retention in care and improve it from time to time. He added that ‘catchment area meetings’ are instrumental in improving retention in care in health facilities within the ‘catchment area’. We believe we have to do it towards a better patient outcome.”

#### Patient information system

This theme summarizes the documentation, updating and sharing of the patient information related to residency, telephone, side-effects of drugs, socio-economic status, and outcomes. The data clerks are at the center of the patient information system.

The health facilities with better retention in care are identified to have both electronic and paper-based patient information. When patients are registered for care they are requested to bring their identification card for their valid address. Moreover, patient’s address is updated whenever the patient comes for their drug refill. The data clerks identify patients “who should come when (every day, every week, today, tomorrow, next week, next month, and so on)” and “who didn’t come for refill when (yesterday, last week, last month, and so on)”. The data clerks then send the list of patients to clinicians and “case managers” for their respective actions.

The data clerk in one of the health facilities said:

“I am carefully documenting all the necessary information about each and every patient before he/she starts treatment. I also update the information whenever the patient comes for medical consultation or drug refill. I am also updating the outcomes of patients. I also share the data with the “case manager” and the clinicians. I have seen that this has helped the “case manager” and the clinicians to provide tailored care. We have learnt that patients with incorrect or no address documented are not traceable once they are lost. Hence, I give due attention to documenting, updating and sharing of information related to patients on ART to the “multi-disciplinary team” in my health facility or with health facilities in the catchment area.”

We summarized and compared the status of implementation of the different interventions that promote retention in care in Table 4 below. One of the health facilities with declining retention in care (NM HC) was a case in point; we found that retention was not considered an important issue; there were very weak retention promoting services. We also found that there were no defaulter tracing and outreach services. There was no community-based organization that provided counseling, care and support services for patients on ART. The health facility didn’t have a cadre, similar to the “case manager”, who coordinated the care of patients. There was not a dedicated data clerk that looked after the patient information system either. As a result, patient information was not updated regularly.

**Table 4:** Comparison of implementation status of interventions for retention in care in health facilities with relatively higher and lower levels of retention in care, 2010

**Level of retention**	**Retention in care promoting activities by health facility and community-based organizations**	**Coordination**	**Patient information system**
**Higher level of retention**	● Consider adherence and retention as the responsibility of each and every cadre involved in the care of patients	● Assign a coordinator called ‘case manager’ responsible for the holistic care of patients	● Assign data clerks that work on the patient information
			● Have both electronic and paper-based patient information system that coordinates, updates and shares patient information regularly with stakeholders
		● Have a mechanism for the coordination and linkage of services	
	● Provide patient tailored adherence and retention-related services		
		● Conduct multi-disciplinary team meetings regularly	
	● Have strong and coordinated defaulter tracing and outreach services		
		● Conduct catchment area meetings regularly	
	● Provide patient tailored and coordinated care and support services		
	● Have community-based organizations that provide counseling, care and support services		
**Lower level of retention**	● Adherence and retention is rarely or not at all considered as the business of each and every cadre involved in the care of patients	● There is no focal person for the coordination of the holistic care of patients	● No dedicated data clerks that work on the patient information
			● There is poor documentation of the patient information
		● There is no mechanism for the coordination and linkage of services	
			● The patient information is not updated and shared regularly with stakeholders
	● There is weak patient tailored adherence and retention-related services		
		● There is weak or no multi-disciplinary team meetings conducted regularly	
	● There is weak and uncoordinated defaulter tracing and outreach services		
		● There are weak or no catchment area meetings conducted regularly	
	● There are few or no community-based organizations that provide counseling, care and support services		

**Figure 2:**
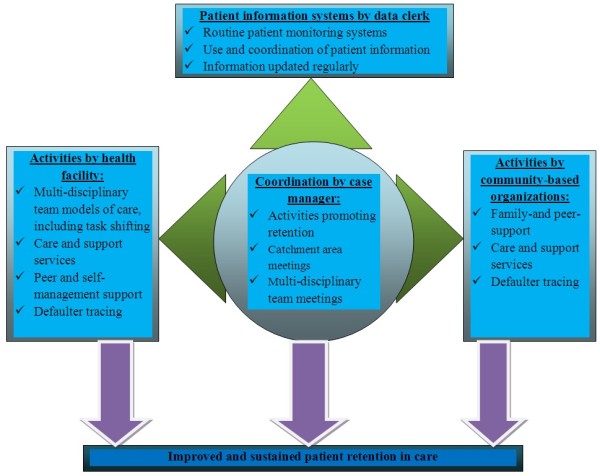
A framework to improve patient retention in care in ART program in Ethiopia.

Based on the themes that emerged from our qualitative study, we developed a framework that comprises the different interventions for improving patient retention in care in ART programs (Figure 2). The framework has got four pillars: activities by the health facility, activities by the community-based organizations, coordination of these activities by the case manager(s), and patient information systems by the data clerk(s).

## Discussion

We found that the baseline characteristics of the patients (CD4-cells count, median age and gender) did not vary significantly across the health facilities (Table 2). However, the level of retention in care was variable across these health facilities: DT HP had the least retention in care (OR = 0.46 (0.35, 0.60), P-value = 0.000) among HPs, and BR HC and NM HC had the least retention in care (OR = 0.44 (0.28, 0.70), p-value = 0.000) among HCs in 2009/2010 (Tables 3). We also found that health facilities which had poor retention in care in 2005/6 were able to catch up with health facilities with better retention in care in 2009/10 (Figure 1). Retention in care dropped between 2005/6 and 2007/8; on the contrary, it had improved between 2007/8 and 2009/10. The variability in levels of retention in care among health facilities was less in 2009/10 than in 2006/7 (Figure 1).

In the earlier phases of the ART scale up in Ethiopia, there was a lot of attention for increasing access to ART. However, there was little attention for retaining patients in care. As a result, there was a fast growing problem of attrition of patients from the ART program in 2005/6-2007/8. It took time before it was recognized that retention in care was a real challenge for the ART program. Later, cognizant of the challenge, a lot of initiatives were implemented to improve retention in care. A “case management program” was thus introduced systematically as a pilot project in very few health facilities in 2007/8. It was afterwards scaled up in a number of health facilities, and decided to be a national program to improve retention in care in the country. A number of health facilities, which were not included in the pilot project, with poor retention in care were able to catch up with health facilities with better retention in care. This was possible as a result of diffusion of best practices through different management practices such as supportive supervisions, review meetings and experience sharing visits among health facilities [6,10,15].

There is a lot of evidence that poor retention in care in resource limited countries is due to factors related to health systems, community and individual patient [16]. In a previous study we found that lack of trust in the services, distance and transport cost, nutrition, opting for alternative traditional medicines, stigma, feeling well, and lack of or inadequate family and community support mechanisms are the main reasons contributing for poor retention in care [5]. These reasons are also described in many studies in developing countries [16].

Our qualitative study identified interventions implemented by health facilities and the community-based organizations to address these barriers for retention in care. Health facilities with better and improving retention in care were found to implement comprehensive packages of interventions. We categorized these interventions into four themes: (1) retention in care promoting activities by the health facility, (2) retention in care promoting activities by the community-based organizations, (3) the coordination of the retention in care promoting activities by the case manager(s), and (4) patient information systems managed by the data clerks(s). These comprehensive packages of interventions were identified to be priorities in high-performing health facilities while they were either low priorities or virtually lacking in low-performing health facilities (Table 4). Based on these themes and sub-themes that emerged from the interviews and FGDs, a framework was developed (Figure 2). The framework consists of four themes presented above and discussed below one by one.

Retention in care promoting activities by the health facility level interventions include: ensuring continuity of care (including consultations, medicines, laboratories, and others); provision of care and support services (including transport, nutrition and other related services); coordination of care within and outside the health facilities; preparedness of health care teams for the needs of patients (including clinical, communication, counseling and related skills); support for patient self-management; implementing models of care that facilitate task shifting and “multi-disciplinary team” approaches (involvement of less qualified health workers and community members); provision of adherence counseling; implementation of defaulter tracing activities; and linkage and coordination with community-based organizations. Health facilities which had high priority and focus on such and related interventions were said to have patients who are more informed, motivated and likely to adhere than the patients in health facilities where these interventions are either not priority interventions or not there at all. Moreover, these health facilities were able to identify patients at risk of poor adherence and/or retention, initiate earlier tracing of patients lost to follow-up.

Retention in care promoting activities by the community level interventions include: presence of community-based organizations which work on awareness creation and stigma reduction; mobilization and coordination of community resources; provision of complementary services like counseling, care and support; presence of family-and peer-support mechanisms; and, coordination of the care of patients with health facilities and other community-based organizations. Such kinds of services are either rarely implemented or not available around the health facilities with relatively low level of retention in care.

Patient information system was also found to be one of the building blocks for improving retention in care in health facilities with better or improving retention in care. Health facilities and community-based organizations have not only patient information and monitoring systems but also the culture of sharing and coordinating the information of patients in their catchment areas. Both health facilities and the community-based organizations have patient information and monitoring systems that enable them to identify patients at risk of poor adherence and/or retention, and take appropriate measures accordingly. The data clerk is at the center of the patient information systems.

In addition to the services and the patient information systems in place, the coordination of the care of patients was also found to be a key building block to improve retention in care. The “case manager(s)” in these health facilities are at the center of coordination of the care of patients. The “case managers” coordinate the patient care given by both health facilities and community-based organizations. Moreover, the “case managers” participate in the “multi-disciplinary team” meetings and “catchment-area” meetings. HIV/AIDS case management is a mode of service delivery for chronic illnesses such as HIV/AIDS, and involves health facilities, community-based organizations, faith-based organizations, governmental and nongovernmental organizations and other community resources. The case management program utilizes a “multi-disciplinary team” approach and a network model around its catchment [17].

Our findings are in line with the findings in other studies which highlight the need for comprehensive packages of interventions to improve retention in care [18]. These interventions were started to be implemented in other chronic diseases such as diabetes and mental illness when a lot of evidence was generated that patients with chronic diseases need services which go beyond health facilities and are delivered at both home and community levels [19,20]. However, health systems in developing countries are basically designed more for acute problems than chronic problems [21]. Moreover, service delivery models in developing countries are labour-intensive and very much relying on physicians, in spite of the lack of highly qualified health workers in these countries [22-24]. It is therefore important that health systems in these countries adapt their health service organisation and delivery in line with the health systems realities of the countries and the life-long needs of chronic patients: delivery models which require less doctor-time and allow rational redistribution of tasks, and respond to the life-long needs of patients [22,25-29].

Moreover, care providers are confronted with transitions (epidemiologic and technologic) that affect the patient-provider relationship with the need to redirect certain care relations towards a more horizontal partnership [30]. The framework in Figure 2 was developed to address the needs of patients with lifelong treatment, the health systems realities of low-income countries, and in line with the chronic care model for patients with chronic illnesses [31,32].

This study has both strengths and weaknesses. The first strength of the study is that it is a mixed methods study that aimed to identify health facilities with relatively better and less retention in care and explore how health facilities with better retention in care were able to achieve that level of retention compared to those health facilities which were not able to do that. This facilitates the design of practical models of care that improve retention in care. The second strength of the study is that it included all tiers of health facilities providing ART including tertiary hospitals, general hospitals and health centers. This can give more robust information than a study that includes only one health facility or health facilities from a limited tier of the health facilities. The third strength of the study is that a framework for improving retention is developed based on the themes that emerged from the interviews and FGDs. The first limitation of this study is that it does not estimate the cost-effectiveness of the interventions implemented by health facilities (and community-based organizations) with better retention in care compared to health facilities with less retention. The second limitation of the study is that it cannot give an estimate of the relative contribution of the different interventions implemented by the health facilities with better and improving retention in care. The third limitation of the study is that the design is not able to assess cause and effect relationship, and there might be other explanatory factors that could not be controlled or accounted for.

This study has both theoretical and practical relevance. The theoretical relevance is that it adds to the body of knowledge for interventions to improve retention in care by developing an evidence-based framework structuring the activities to improve patient retention in a resource-limited setting. The practical relevance of the study is that it is addressing the real challenge of many ART programs which are striving hard to manage and sustain them towards universal access to care and treatment services. Hence, the findings from this study will help policy makers, program managers and implementers to design and implement interventions towards better retention in care and improved patient outcomes.

## Conclusion

Retention in care in ART program is variable across health facilities in Ethiopia. Some health facilities which had low levels of retention in care at the beginning of the ART delivery were able to improve and catch up with those health facilities which had had relatively higher levels of retention since the earlier phases of the ART delivery. Compared to health facilities with poor retention in care, health facilities with higher and improving retention in care were found to implement more frequently a comprehensive package of interventions targeting adherence and retention: retention promoting activities by the health facility, retention promoting activities by the community-based organizations, coordination of these activities by the case manager(s), and patient information systems by the data clerk(s). We therefore developed a framework, based on these four pillars for improving retention in care. We thus recommend that health facilities with low levels of retention in care start to implement this comprehensive package of interventions, monitor and evaluate their effectiveness, and adapt them to their contexts.

## Competing interests

The authors declare that they have no competing interests.

## Authors’ contributions

YA: conceived the study, coordinated and participated in the data collection, conducted the data analysis and interpretation, developed the first draft, and revised subsequent drafts. LL: commented on successive drafts. EW: commented on successive drafts. FR: commented on successive drafts. KR: commented on successive drafts. WVD: advised on the conception of the study idea, data analysis and interpretation, commented on successive drafts. All authors approved the final version for submission.

## Pre-publication history

The pre-publication history for this paper can be accessed here:

http://www.biomedcentral.com/1472-6963/14/45/prepub
